# Lipoprotein glomerulopathy resulting from compound heterogeneous mutations of *APOE* gene: A case report

**DOI:** 10.1097/MD.0000000000028718

**Published:** 2022-02-04

**Authors:** Yunsi Li, Jin Chen, Yurong Zou, Wei Wang, Guisen Li

**Affiliations:** Renal Department and Institute of Nephrology, Sichuan Provincial People's Hospital, School of Medicine, University of Electronic Science and Technology of China, Sichuan Clinical Research Center for Kidney Diseases, Chengdu, China.

**Keywords:** APOE, hyperlipidemia, lipoprotein glomerulopathy, nephrotic syndrome

## Abstract

**Rationale:**

Lipoprotein glomerulopathy (LPG) is a rare glomerular disease characterized by the deposition of lipoprotein thrombi in glomerular capillaries. The disease is characterized by proteinuria, progressive renal failure, and characteristic lipoprotein thrombosis in glomerular capillaries. Rare mutations in the apolipoprotein E (*APOE*) gene mainly contribute to disease pathogenesis.

**Patient concerns:**

A 28-year-old man presented with severe proteinuria and hyperlipidemia. The patient was treated with a full dose of prednisone for 2 months and then combined with leflunomide 20 mg daily for 20 days; however, his edema continued to worsen.

**Diagnosis:**

The patient was diagnosed LPG by laboratory examination and renal biopsy.

**Interventions:**

The patient was treated with atorvastatin (20 mg) combined with irbesartan (75 mg) once a day.

**Outcomes:**

The patient's lipidaemia and proteinuria were significantly reduced. Genetic testing showed that the patient carried compound heterozygous mutations in *APOE*. *The APOE* gene was inherited from her mother and father. Parents with a heterogeneous mutation had normal kidney function without proteinuria.

**Lessons:**

Usually, a single mutation in *APOE* can lead to the pathogenesis of LPG. This case shows that LPG could result from compound heterogeneous mutations of *the APOE* gene inherited from his mother and father. Intensive lipid-lowering combined with RASIs is effective in patients with LPG. Early renal biopsy and genetic mutation detection can avoid the unnecessary use of glucocorticoids and immunosuppressants.

## 1 Introduction

Lipoprotein glomerulopathy (LPG) is a rare genetic metabolic disease closely related to type III hyperlipidemia.^[1]^

As early as 1987, Faragianag et al described these manifestations in their review of lipid nephropathy.^[2]^ In 1989, Saito et al first proposed the concept of lipoprotein nephropathy, named LPG,^[3]^ and described its clinical and pathological characteristics. Then, it was reported that LPG can result from *APOE* gene mutation.^[4–8]^

Up-to-date, ∼150 adult cases of LPG have been reported globally, most of which were in Asia. Mutation of one locus of *the APOE* gene can lead to LPG. Globally, *APOE* Kyoto is the major LPG mutant.^[9]^ Here, we report the case of a patient who carried two heterozygous variants of *the APOE* gene and his clinical characteristics.

## 2 Case presentation

A 28-year-old man presented to our hospital with proteinuria and edema of both lower extremities on March 23, 2021. He was diagnosed with NS 6 months ago at a local hospital and presented with proteinuria (4+), hyperlipidemia (serum total cholesterol, 7.09 mmol/L, low-density lipoprotein cholesterol [LDL-C], 6.39 mmol/L), and normal serum albumin level. The patient was treated with prednisone 60 mg/day for 2 months. His edema disappeared under symptomatic treatment; however, his proteinuria did not achieve remission, and the dosage of prednisone was tapered rapidly. Three months ago, his lower limb edema had recurred, and he was administered symptomatic treatment.

**Figure 1:**
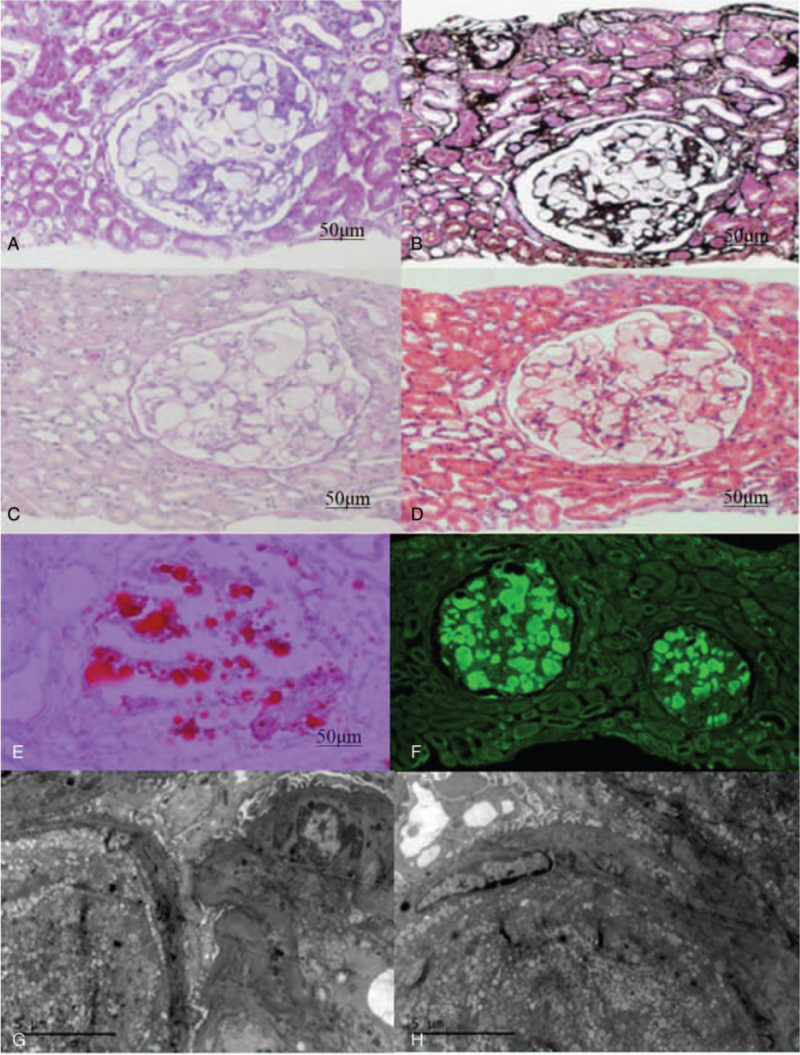
Renal pathologic manifestations. (A) The dilated capillary loops are occupied by red light-staining substances (Masson). (B) The dilated glomerular capillaries filled with thrombi (PASM). (C) The dilated capillary loops are occupied by lipoprotein thrombi (PAS). (D) The dilated capillary loops are occupied by lipoprotein thrombi (HE). (E) Positive Oil-Red-O staining in the lipoprotein thrombi. (F) Immunofluorescence staining shows *APOE*-positive deposits, particularly within the glomeruli(×400). (G and H) electronic microscopy shows lipoprotein-like protein deposition in glomerular capillaries.

Because of persistent heavy proteinuria, he had received prednisone combined with leflunomide 20 mg daily, for 20 days previously. Upon admission to our hospital, a physical examination revealed mild pitting edema in both lower extremities. No obvious abnormal signs in the heart or lung were found on physical examination. His blood pressure on admission was 154/106 mm Hg. Urine test results showed proteinuria of 7.689 g/24 h. Laboratory analysis showed that he had hyperlipidemia (total cholesterol 6.99 mmol/L, LDL-C 3.78 mmol/L, triglyceride [TG] 2.51 mmol/L), decreased serum albumin 26 g/L, as well as normal serum creatinine 68 μmol/L and estimated glomerular filtration rate 123.54 mL/min. A renal biopsy was performed. Light microscopy revealed lipoprotein thrombi in the glomeruli (Fig. 1A–D). Electron microscopy showed lipoprotein-like deposition in the glomerular capillaries (Fig. 1G and H). We then stained the renal biopsy with Oil Red O, and the staining was strongly positive (Fig. 1E). Immunofluorescence staining revealed *APOE*-positive deposits, particularly within the glomeruli (Fig. 1F).

### 2.1 Mutational analyses of the *APOE* gene

**Figure 2:**
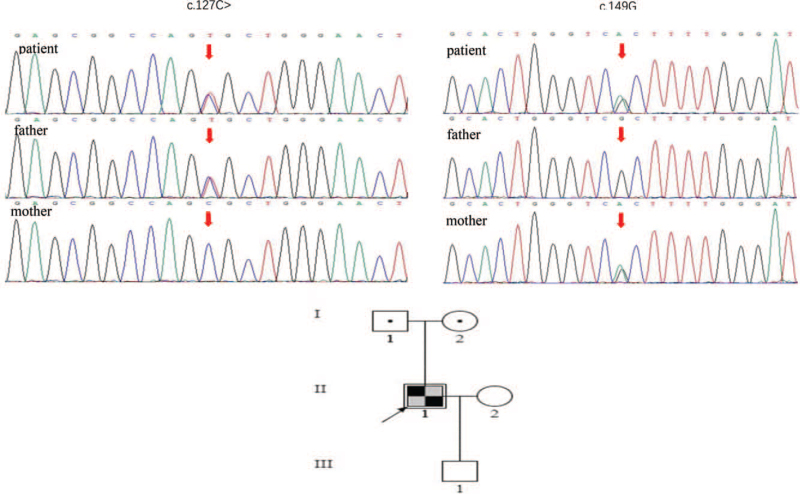
Analysis of AOPE gene mutation in the family: sequencing analysis demonstrating the detection of c.127C > T(p.Arg43Cys) mutation and c.149G > A (p.Arg50His) mutation in exon 7 of the *APOE* gene. Family tree:Parents were normal phenotype, but the patient is presented with LPG.

We performed genetic testing using next-generation sequencing in this family. The *APOE* gene mutation for c.127C > T (p.Arg43Cys) in the father was a missense heterozygous mutation, named the *APOE* Kyoto mutation (Fig. 2). The *APOE* gene mutation c.149G > A (p.Arg50His) in his mother was detected and was another missense heterozygous mutation with a suspected pathogenic variant (Fig. 2). This is a novel mutation in the *APOE* gene associated with LPG. Both parents had a normal renal phenotype without proteinuria or increased serum creatinine levels. The patient carried two heterozygous mutations in the *APOE* gene and presented with compound heterozygous mutations (Fig. 2).

### 2.2 Clinical course

**Figure 3:**
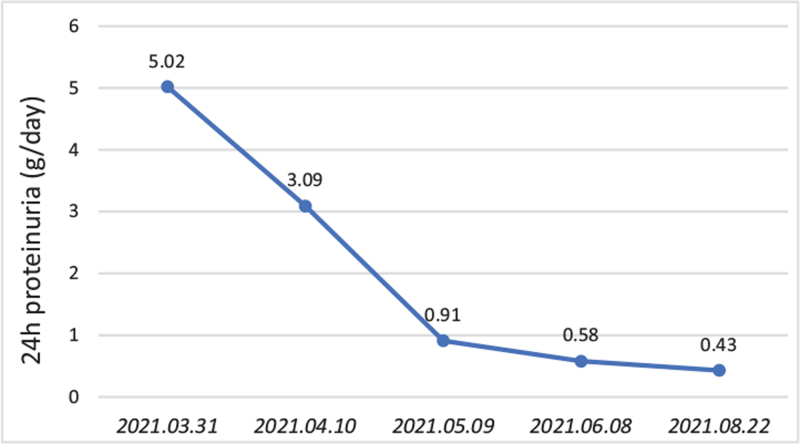
The change of patient's proteinuria within 5 months.

The patient was treated with atorvastatin 20 mg and irbesartan 75 mg once a day. The patient was followed up regularly. After about 6 weeks of treatment, the patient's proteinuria decreased significantly to 0.43 g/24 h and serum albumin increased to 47.6 g/L (Fig. 3).

## 3 Discussion

LPG is an autosomal recessive genetic disorder. The core problem in lipoprotein nephropathy is an abnormality of apolipoprotein E (apoE) coded by *APOE* gene. ApoE is a glycoprotein comprising 299 amino acids. *ApoE* monomers have three phenotypes, E2, E3, and E4, corresponding to genotypes ε2, ε3, and ε4, respectively. The *apoE* of healthy individuals is composed of two monomers: *apoE*3/3. The *apoE* phenotype in patients with lipoprotein nephropathy is mainly E2/3.^[10–15]^ In China, mutations in *apoE* Chengdu (p.L173P),^[16]^*apoE* Guangzhou (p.Rl50P),^[17]^*apoE* Shenzhen (p.R150C),^[18]^*apoE* Hong Kong (p.D230Y),^[19]^ and *apoE* Kyoto^[20]^ have been reported. The *Kyoto APOE* mutation is a common mutation in LPG, and functional studies have shown that this variant affects normal protein function (PS3).^[21]^ The previous reports have demonstrated that a heterozygous form of the *APOE* Kyoto mutation was detected in patients with LPG, but some pedigree members carrying the variant had no corresponding clinical manifestations.^[8]^ Another report also showed that 28 asymptomatic carriers indicated incomplete penetrance of the Kyoto mutation; however, the detailed mechanisms of incomplete penetrance remain unknown.^[20]^

LPG diagnosis depends on clinical, pathological, and laboratory surveys.^[22]^ The Angiotensin-converting enzyme inhibitors, fenofibrate, and apheresis (e.g., immunoadsorption using protein A columns) have been demonstrated to improve the outcome of patients with LPG.^[21]^ But there is insufficient data to highlight the roles of different heterozygous forms of *APOE* in diagnosis, treatment, prognosis, and pregnancy counseling.

The interesting point of this case is that the patient carried compound heterozygous mutations in *APOE* gene inherited respectively from his parents. However, his parents had a normal renal phenotype without proteinuria or increased serum creatinine levels. However, the patient carrying these two heterozygous mutations developed LPG. This could be a cumulative effect of the two heterozygous mutations. A novel mutation associated with LPG, c.149G > A (p.Arg50His), was detected in his mother but was not reported in patients with LPG. This variant has been detected in patients with familial hypercholesterolemia and early onset coronary heart disease. After treatment with atorvastatin 20 mg daily for 6 weeks, the patient's proteinuria was significantly reduced and the serum albumin level increased to the normal range. In this case, compound heterozygous mutations of the *APOE* gene contributed to the pathogenesis of LPG. This case provides a new perspective for investigating the pathogenesis of LPG.

## Acknowledgments

We would like to thank the patient who participated the research.

## Author contributions

**Investigation:** Jin Chen, Yurong Zou, Wei Wang.

**Supervision:** Guisen Li.

**Writing – original draft:** Yunsi Li, Guisen Li.

**Writing – review & editing:** Guisen Li.
